# Self-rated health and quality of life in female sex workers with substance use disorders in Tehran, Iran

**DOI:** 10.1186/s12905-023-02552-4

**Published:** 2023-08-01

**Authors:** Effat Merghati Khoei, Zahed Rezaei, Arash Parvari, Jeffrey E. Korte

**Affiliations:** 1 Sexual Health Promotion, The Iranian Center for Addiction Studies (INCAS), Tehran University of Medical Sciences, Tehran, Iran; 2 Sexual & Family Health Division in the Brain and Spinal Cord Injury Research Center (BASIR), Neuroscience Institute, Tehran University of Medical Sciences, Tehran, Iran; 3 Asadabad School of Medical Sciences, Asadabad, Iran; 4 Department of Epidemiology and Biostatistics, School of Public Health, Tehran University of Medical Sciences, Tehran, Iran; 5 Department of Public Health Sciences, School of Medicine, Medical University of South Carolina, Charleston, United States of America

**Keywords:** Female sex worker, Self-rated health, Quality of life, Substance Use Disorder, Iran

## Abstract

**Background:**

While self-rated health (SRH) and quality of life (QoL) has been associated with substance use disorders (SUDs) in sex-working populations, little is known about this association in Iran. This study aimed to assess QoL and SRH in Iranian female sex workers (FSWs) in Tehran.

**Method:**

FSWs were recruited using convenience sampling methods from substance abuse treatment centers in Tehran that exclusively provided services for women. Participants completed an interviewer-administered demographic questionnaire in Persian and the Iranian version of the Short Form Health Survey (SF-36). Descriptive analyses, means and standard deviations; frequency and percentages, t-test and one-way ANOVA, and Chi-square tests were utilized to analyze the data.

**Results:**

The mean age among 161 participants clinically diagnosed with SUD was 34.09 years (SD 7.97; range: 18–57). The total mean QoL score was 41.03 (SD: 12.92). The highest and lowest mean scores were observed in the physical functioning (52.23) and role emotional (26.64) dimensions, respectively. Significant differences (p < 0.05) in QoL were observed according to education and marital status, and the average QoL score was lower in women who reported permanent marriages and women who were illiterate. The average score of QoL was significantly higher in employed women. Overall, 51.6% of the women rated their health as sub-optimal, with divorced participants and women who were illiterate more likely to rate their health as sub-optimal (p < 0.05).

**Conclusion:**

Results emphasize the need for mental, physical, and sexual health screening and gender-specific interventions to improve QoL in this population. Further investigation may elucidate the consequences of poor SRH and QoL on SUD treatment adherence, sexual risk behavior, and morbidity and mortality in FSWs.

## Introduction

Quality of life (QoL (and health status of female sex workers (FSWs) is considered a major public health concern and low- and middle-income countries (LMICs) are no exception. A systematic review and meta-analysis revealed that female sex workers (FSWs) from LMICs suffer from poverty, low education, violence, alcohol and drug use, human immunodeficiency virus (HIV), and stigma and discrimination. The authors found that poor mental health is highly prevalent among this population due to the identified determinants [1].

If two criminalized and marginalized activities such as sex work and SUDS come together, women are more at risk. Psychosocial dysfunction such as lifetime trauma, suicide, social conflict, injecting drug use, and having co-morbid medical and psychiatric impairment have been reported [2, 3]. Subsequently, they should be the center of focus of researchers and professionals in the field of sexual risk behaviors and drug issues. With these scientific reports in hand, it is not surprising that vulnerable women evaluate their health status as poorer than other members of society [4]. As a leading health indicator, SRH or self-assessed health is used to explain one’s overall health status, social functioning, and QoL [5]. It is possible that women with SUDs have additional health-related concerns, and that their QoL may be more impaired due to the impact of sex work on their self-perceived health status, yet limited research has explored this.

We invoke syndemic theory to explain the potential for health problems among sex workers with or without SUDs. A syndemic is “two or more afflictions, interacting synergistically, contributing to excess burden of disease in a population” [6]. Scholars emphasize the situational factors linked to episodes of risky behaviors among at-risk or high-risk populations [7]. It is important to understand situational factors linked to selling sex and SUDs among sex workers, who are disproportionately affected by sexual risk behaviors, sexually transmitted infections (STIs), discrimination, violence, and social traumas in Iran [8–14]. Iranian sex workers with SUDs who are not educated, employed, or socially supported, are more likely to experience poor life quality and poorer general health outcomes [15], similar to other vulnerable populations [4, 16]. The quality of life of a sex worker with SUDs will differ on average from a sex worker without SUD. It has been shown that the most important dimension of a sex worker’s life depends on her damaged social interactions [17], and this is extremely problematic among Iranian sex workers with SUDs. Damaged social interactions can be chained with some of the consequences such as socioeconomic hardship, increased stigma and violence, and breached human rights; the challenges a sex worker may experience where selling sex is culturally intolerable, criminalized, and socially banned [18, 19].

Although so much data exist about the Iranian FSWs and women with SUDs; nevertheless they leave a gap in an accurate assessment including whether SUDs and selling sex additively and interactively contribute to health indicators (i.e. QoL and SRH). In this regard and in trying to attain effective interventions in solving the health problems of sex workers with SUDs, we decided to search for scientific evidence on their quality of life and health status by conducting this research. In this study, we hypothesized that a syndemic of co-occurring sex work and SUDs are additive and associated with poor QoL and inadequate SRH of Iranian FSWs.

## Methods

### Design and data collection

This cross-sectional study was carried out from 2018 to 2020 to assess the quality of life and self-rated health in Iranian FSWs. The present study was carried out on 161 Iranian FSWs with SUDs recruited from six substance abuse treatment centers in Tehran that exclusively provided services for women. Participants were recruited using convenience sampling among all women enrolled/registered at these centers in Tehran, the capital of Iran. The inclusion criteria were as follows: (a) women aged over 15 years, (b) single or married (i.e. permanent or temporary); in the *Shia* Islamic contexts, marriage is defined in two forms: fixed-term/temporary/Pleasure marriage and the long-term/permanent/conventional. The main difference is that the temporary marriage lasts only for a specified period, so-called *sigheh* (an Arabic name concept), and the man and woman will become strangers to each other after the expiration date without divorce. (c) sexually active (oral, anal, or vaginal) with any form of the sexual partner (man/woman or both) in the last six months, (d) history of substance use and currently in treatment (with or without pharmacotherapy), (e) willing to provide voluntary informed consent. This study was approved by the Iranian Research Center for HIV/AIDS, (Ethic Approval Code: 92-01-55-19799) and women were remunerated including underwear, a sanitary package, and light food for their participation.

The data were collected on-site in each clinic by trained interviewers in the Persian language using a two-part questionnaire. The first part contained demographic questions including age, marital status, education, and employment status. The second part of the questionnaire consisted of the Iranian version of the Short Form Health Survey (SF-36). The SF-36 consists of 36 questions measuring eight dimensions of quality of life. These dimensions are physical functioning, role impairment due to physical health/role physical, role impairment due to emotional health/role emotional, energy and fatigue/vitality, mental health, social functioning, body pain, and general health. Two other general subscales are achieved by integrating the subscales known as Physical Component Summary (PCS) and Mental Component Summary (MCS). The total score from each dimension ranges from 0 to 100, with higher scores indicating higher QoL. The psychometric properties of the Iranian version of SF-36 are well documented in Iran [20]. Although the SRH has been introduced as a valid and reliable tool in health surveys among community-dwelling older adults [21], we employed the standard SF-36 to measure SRH. The question was: “In general, how would you rate your health?” with the response categories “excellent”, “very good”, “good”, “fair”, and “poor”. For the analyses, the variable was dichotomized into optimal SRH (“excellent”, “very good” and “good”) and sub-optimal SRH (“fair” and “poor”).

### Data analysis

Descriptive analyses were conducted to calculate the distribution of demographic variables, QoL and SRH. Data related to continuous variables were reported as means and standard deviations; categorical data were reported as frequencies and percentages. Differences of QoL in categorical variables were examined by *t*-test and one-way ANOVA. Chi-square tests were used to compare SRH between different demographic groups. Data were analyzed by SPSS statistical software (Version 24.0) with a statistical significance set at p < 0.05.

## Results

The mean age of respondents (n = 161) was 34.09 years (SD 7.97; range: 18–57). Few women were single (21.1%; n = 34) and 41.6% were divorced (n = 67). In addition to sex work, most of them had no other jobs or sources of income (80.1%). (Table ​1).

**Table 1:** Characteristics of female sex workers with SUD attending women-specific drug treatment clinics/centers in Tehran (N = 161)

Variable	Category	Number	Percentage (%)
**Age**	18–30	60	37.3
	31–40	70	43.5
	Above 40	31	19.3
**Marital Status**	Single (never married)	34	21.1
	Married (permanent marriage)	19	11.8
	Married (temporary marriage)	22	13.7
	Divorced	67	41.6
	Widowed	19	11.8
**Education Level**	Illiterate	13	8.1
	Elementary	47	29.2
	Secondary and high school	59	36.6
	Diploma	29	18
	Associate degree and bachelor degree	13	8.1
**Employment**	Yes	32	19.9
	No	129	80.1

Table 2 presents the scores of all subscales of the QoL among the FSWs. The total mean score of the QoL was 41.03 (SD: 12.92). The highest and lowest mean scores were observed in the physical functioning (52.23) and role emotional (26.64) dimensions, respectively.

**Table 2:** Quality of life dimensions among female sex workers (N = 161)

QoL dimensions	Observed range	Mean	SD
General Health	15–90	40.80	14.56
Physical functioning	0-100	52.23	26.99
Role physical	0-100	29.68	34.35
Bodily pain	0-100	44.05	22.42
Social functioning	12.50–100	51.32	19.84
Mental health	4–80	39.23	11.76
Vitality	10–95	44.28	14.95
Role emotional	0-100	26.64	21.35
The total score of QoL	9.88–79.38	41.03	12.92

One-way ANOVA analysis showed statistically significant differences (p < 0.05) according to education and marital status for QoL scores, with average scores lower in women reporting permanent marriage and women who were illiterate. The t-test showed that the average QoL score was significantly higher in employed than in unemployed women and this difference was statistically significant (p = 0.03) (Table 3).

**Table 3:** Associations between QoL and demographic characteristics among female sex workers (n = 161)

Variable	Category	The total score of QoL			P value
		Mean	SD		
**Age**	18–30	40.94	12.05		0.36*
	31–40	39.87	13.39		
	Above 40	43.82	13.46		
**Marital Status**	Single (never married)	50.06	13.95		< 0.001*
	Married (permanent marriage)	32.67	5.67		
	Married (temporary marriage)	39.33	11.31		
	Divorced	40.05	11.73		
	Widowed	38.46	14.16		
**Education Level**	Illiterate	33.93	7.45		0.02*
	Elementary	39.15	8.91		
	Secondary and high school	43.63	12.82		
	Diploma	38.82	17.64		
	Associate degree and bachelor degree	48.04	13.40		
**Employment**	Yes	45.39	14.53		0.03**
	No	39.98	12.33		

*One-way ANOVA

**Independent t-test

Overall, 51.6% of the respondents rated their health as sub-optimal. The rate of sub-optimal SRH varied significantly between some subgroups. It was significantly higher among divorced and illiterate groups (p > 0.05) (Table 4).

**Table 4:** Association between SRH and demographic characteristics among female sex workers (n = 161)

Variable	Category	SRH N (%)		P value
		Optimal 78 (48.4)	Suboptimal 83 (51.6)	
**Age**	18–30	29 (48.3)	31 (51.7)	0.49
	31–40	30 (42.9)	40 (57.1)	
	Above 40	11(35.5)	20 (64.5)	
**Marital Status**	Single (never married)	22 (64.7)	12 (35.3)	0.01
	Married (permanent marriage)	11 (57.9)	8 (42.1)	
	Married (temporary marriage)	10 (45.5)	12 (54.5)	
	Divorced	20 (29.9)	47 (70.1)	
	Widowed	6 (31.6)	13 (68.4)	
**Education Level**	Illiterate	2 (15.4)	11 (84.6)	0.02
	Elementary	16 (34)	31 (66)	
	Secondary and high school	27 (45.8)	32 (54.2)	
	Diploma	16 (55.2)	13 (44.8)	
	Associate degree and bachelor degree	9 (69.2)	4 (30.8)	
**Employment**	Yes	16 (50)	16 (50)	0.84
	No	62 (48.1)	67 (51.9)	

## Discussion

Our findings suggest that poor QoL coincides with health problems in Iranian FSW with SUD, taking place in a context of social marginalization. These findings are in line with Iranian research reports [15] as well as others [22]. Our findings emphasize that understanding Iranian FSWs’ SRH status as well as their low QoL is important. Undoubtedly, as citizens, they have the right to good health and quality of life. On the other hand, their good health directly affects the health of their clients, with clear implications for public health. In a study of FSW in the United States, Murphy [22] found that the social and economic conditions surrounding women can hold them in sex work, damaging their health and quality of life. In Iran, in addition to the social exclusion of this group of women, FSWs themselves seem to play an important role in their isolation. In terms of isolation, our experience with Iranian FSWs shows that they do not consider good health to be their right, and do not strive for a high quality of life, while they must try as other citizens to receive health services and live better lives. The possible explanation for their isolation can be the Iranian culture that extremely stigmatized this population and force them to move underground. Using a phenomenological inquiry, the challenges and experiences of Iranian FSWs were explored [19]. The majority of SWs experienced violence due to poor socio-economic situation; they suffer poor and risky health; social exclusion due to dissociated relations; objectification, and lack of social and legal support. In another qualitative study with 24 FSWs, Karamouzian et al. (2012) introduced a conceptual framework that describes FSWs’ experiences of “vulnerability before entering sex work, their initiating sex work and their reasons for staying in the business” (pp.10). Iranian FSWs move through the different phases of sex work based on various factors related to poverty, coercion, choice, and opportunity. These factors make them vulnerable and cause poor economic status. Furthermore, their misperceptions about sex work as a job, limited life opportunities, and lack of family support affect their health status and QoL [23].

About half of the participants )51.6%( rated their health as sub-optimal.We could not find similar reports to support this finding. Similar to others [24–26], our participants seem to use different health-related frames of reference when making their health judgments. The prevalence of poor SRH in the general female population in Iran has been observed between 13.5% and 37.8% [27–30]. Ultimately comparing the general female population with FSWs could be biased since FSWs and other women may differ in how they subjectively rate their health.

Our results indicate that ratings of sub-optimal SRH were significantly higher among women who were divorced and illiterate women. Persistent educational disparities in self-rated health have been well documented over time [31]. Contrary to our findings, Lamidi (2020) showed reduced odds of fair or poor self-assessed health with increasing educational achievement among married, cohabiting, previously-married, and never-married adults. Sub-optimal SRH among FSWs who were illiterate may reflect differences in economic opportunities at different levels of education among sex workers in Iran. Lamidi’s findings show an overall health decline among single (previously married and never married) adults in the general population, supporting the lower SRH observed among divorcees in our study. However, results from our study and Lamidi’s study may not be directly comparable due to important differences in the two study populations.

### Limitations

Although we believe that 161 Iranian sex workers with SUDs are still a good size to study, however, we still consider it a limitation of our study. First, these women are an underground population and we had the problem of having limited access to them. Second, we had limited funding and had to complete the project with the minimum statistically acceptable sample. We acknowledge a need for a future (e.g., a longitudinal study) to answer this research problem and overcome the possible negative impact of time or funding constraints on the study. With 161 participants it was difficult to identify substantial relationships in the data or answer ‘why’ questions. To draw valid conclusions and more precise results in the field of our interest, we recommend a larger sample or conducting a study with a different methodology (i.e. qualitative studies).

## Conclusion

Iranian FSWs with SUDs in the current study reported poor SRH and low QoL. Based on our findings, it is necessary to break the cycle of risky health behaviors, substance use, sex work, and reduced quality of life in vulnerable women to improve their QoL and overall health [32] and minimize the adverse impacts of sex work on their quality of life and health. Our results emphasize the need for physical, mental, and sexual health screening and further development of gender-specific interventions to improve QoL in this population. An important area for future research will be to understand the differences between FSWs with and without SUDs in risky sexual behaviors, QoL and SRH. Further investigation may elucidate the consequences of poor SRH and QoL on SUD treatment adherence, sexual risk behavior, and morbidity and mortality in FSWs.

## Data Availability

The data are available upon request to the corresponding author after signing appropriate documents in line with ethical application and the decision of the Ethics Committee.

## Abbreviations

SRH: Self-Rated Health
QoL: Quality of Life
SUDs: Substance Use Disorders
FSW: Female Sex Workers
LMICs: low- and middle-income countries
HIV: human immunodeficiency virus
STIs: sexually transmitted infections
PCS: Physical Component Summary
MCS: Mental Component Summary
